# Special issue “Facets of Global Health: Globalisation, Equity, Impact and Action”

**DOI:** 10.3402/gha.v7.23696

**Published:** 2014-02-13

**Authors:** Tewabech Bishaw

The collection of articles in this special issue provides a broad overview on various facets of global health, and it attempts to establish the link between globalisation and its manifestation and impact on the regional and local levels. The idea for this collection was first conceived during the 13th World Congress on Public Health, held in Addis Ababa, Ethiopia, 23–27 April 2012, and evolved out of deliberations regarding the lack of comprehensive, dedicated and affordable textbooks for teaching global health. In the aftermath of the World Congress, a group was established to develop a first collection of articles for such textbooks. This special issue is the result of a collaboration that brought together representatives from the World Federation of Public Health Associations (WFPHA), the African Federation of Public Health Associations (AFPHA) and the Department of International Health at Maastricht University. Because global health is an emerging multisectoral field linking (among others) health, trade, governance and foreign policies, development and humanitarian aid, the topics that can be covered in one special issue are necessarily limited. This collection of articles focuses therefore on aspects of governance, international relations and global health policies and on the role of health systems and health systems design including health system reform, primary care and the globalized health workforce. Further facets that are discussed include global health ethics and education. Other important aspects such as global health and global environmental change or global health and urbanisation have not been covered in this special issue but all articles intend to provide an up-to-date overview of the rapidly growing global health literature.

## Overview on Content

The introductory article by Laaser and Brand (1) traces the current state of global health and emerging health issues in the 21st century. Kickbusch and Szabo (2) elaborate on the distinguished differences between global health governance, global governance for health, and governance for global health, and they argue that a clear understanding of these different dimensions for global health will be necessary. Two articles cover the issues of development aid strategies and the current status of the United Nations’ Millennium Development Goals (MDGs) debate. Vassall et al. (3) map European Union aid flows and discuss the obvious imbalances in targeting this aid. Lomazzi et al. (4) analyse the possible pathways towards the post-MDGs and the foreseeable difficulties in adjusting international aid in this process to the contemporary challenges. Aluttis et al. (5) analyse the cumbersome path towards a coordinated European global health strategy from an agenda-setting perspective. Krumeich and Meershoek (6) identify major challenges for social determinants of health (SDH) frameworks in forming concrete policies and interventions at the local, national and global levels. A comprehensive review by Stapleton et al. (7) discusses the moral significance of health in connection with geopolitical boundaries and refers to a selection of relevant ethical theories and practical conflicts. With a focus on the corresponding regional and national levels, Senkugobe et al. (8) illustrate how health systems can be strengthened by health sector reforms. Rao and Pilot (9) discuss the importance of primary care and provide an overview of the current debate. The health workforce crisis – in light of dissolving borders and global markets – is discussed by Aluttis et al. (10) based on a literature review. In the final article, Bjegovic et al. (11) provide good and best practice examples of how health education and research can contribute to the strengthening of health systems.

The editors are grateful for the contributions of authors from different regions of the world, and they consider this special issue a further step in promoting the debate on how to structure the advancing globalisation and its impact on population health. Hopefully, the assembled papers may serve as a further encouragement for teaching and researching global health. The editors thank Prof. Mark Rosenberg (Queens University, Kingston, Ontario) and Prof. Stephen Matlin (Imperial College, London) for their sound reviews of all manuscripts and for their valuable advice. Financial support from the Department of International Health, Maastricht University, is gratefully acknowledged.

## Editors

Tewabech Bishaw, African Federation of Public Health Associations (AFPHA)

Thomas Krafft, European Academic Global Health Alliance (EAGHA)

Ulrich Laaser, World Federation of Public Health Associations (WFPHA)

## Associate Editors

Christoph Aluttis, Department of International Health, Maastricht University

Helmut Brand, Department of International Health, Maastricht University

30 January 2014

Maastricht, The Netherlands
